# Practitioners' Bookshelf

**Published:** 1892-01-02

**Authors:** 


### PRACTITIONERS' BOOKSHELF.

### PRACTICAL DISPENSING.*

This work is intended chiefly for the use of students of medicine and pharmacy, but might be read with profit by members of the medical profession. It is essentially a practical treatise on the art of dispensing, and contains a great deal of useful information and valuable advice on the various methods of combining and dispensing drugs. The general arrangement of the work is excellent. The section on "mixtures" includes most of the difficulties likely to be encountered by the dispenser in the combination of drugs in solution. These difficulties are illustrated by prescriptions, and the methods of avoiding them, and of presenting the various combinations of drugs in the most pleasing form, are explained in a clear, concise, and scientific manner. A list of the principal drugs and their chief incompatibilities is included in this section. The section on pill making and pill coating is exceptionally good, and abounds with useful and practical hints. An appendix is added, which describes the method of making poultices, and a variety of foods suitable for invalids. This is a very useful addition, and might, with advantage, be enlarged. A list of a number of drugs, with their solubility in alcohol and water, completes the appendix. This book ought to prove of considerable service to the student of pharmacy, and should be used to assist, and not to replace, his practical experience.

### PHOTOGRAPHIC GROUP OF THE BACTERIOLOGICAL SECTION OF THE CONGRESS OF HYGIENE, LONDON, AUGUST, 1891.

Messrs. Berraud and Sons (Oxford Street) have contributed an interesting memento of the Congress of Hygiene held in London last August, by producing a most excellent group of the bacteriological section of the meeting. As a photograph it is excellent, and as a picture it contains some capital studies in physiognomy. The portraits are characteristic, and good as likenesses. As a practical nation we cannot, alas, pride ourselves numerically on our contribution to this picture. Nor when we come to reckon up our share in the building up of this new and practical science is our total large. Sir Joseph Lister, however, is a host in himself, and even amongst foreigners he stands out a veritable giant; the co-partners with him in this new science, namely, Pasteur and Koch, by their absence from this group, deprive it of its entirely representative character, but apart from these two absentees, the group contains the chief bacteriological "savants" in Europe. Metchmkoff, of phagocytic fame; Roux, Pasteur's fellow worker; Arloing, Hueppe, and a whole host of lesser luminaries. Kitcsato lends interest to the picture, as being Japan's enterprising representative, and the author of some excellent work on tetanus. Hankin, of Cambridge, will be of interest to Englishmen, as a young man of great promise, and young as he is, possessing a world-wide reputation. In a group where all the likenesses are first-rate, the genial face of Sir Joseph Lister is good to look upon, and in Professor Burden Sanderson the philosopher stands revealed.

